# Introducing "*Frontiers in Zoology*"

**DOI:** 10.1186/1742-9994-1-1

**Published:** 2004-09-29

**Authors:** Jürgen Heinze, Diethard Tautz

**Affiliations:** 1 Biologie I, Universität Regensburg, D-93040 Regensburg, Germany; 2 Institute for Genetics, Weyertal 121, D-50931 Cologne, Germany

## Abstract

As a biological discipline, zoology has one of the longest histories. Today it occasionally appears as though, due to the rapid expansion of life sciences, zoology has been replaced by more or less independent sub-disciplines amongst which exchange is often sparse. However, the recent advance of molecular methodology into "classical" fields of biology, and the development of theories that can explain phenomena on different levels of organisation, has led to a re-integration of zoological disciplines promoting a broader than usual approach to zoological questions. Zoology has re-emerged as an integrative discipline encompassing the most diverse aspects of animal life, from the level of the gene to the level of the ecosystem.

The new journal *Frontiers in Zoology* is the first Open Access journal focussing on zoology as a whole. It aims to represent and re-unite the various disciplines that look at animal life from different perspectives and at providing the basis for a comprehensive understanding of zoological phenomena on all levels of analysis. *Frontiers in Zoology* provides a unique opportunity to publish high quality research and reviews on zoological issues that will be internationally accessible to any reader at no cost.

## The revival of integrative zoology

Zoology has a long history of more than 2000 years and is one of the natural sciences attracting most public attention. Nevertheless, as a scientific field, zoology occasionally is considered to be old-fashioned and in danger of being replaced by narrower sub-disciplines that focus on a few important and in vogue aspects of animal biology. The remarkable success of such specialized approaches has in the last decades promoted the breakdown of zoology into a number of autonomous research areas, some focussing on molecules and cells and others on whole organisms or ecosystems, each with its own specialized journals and with little exchange of concepts and empirical information between them.

The advance of molecular methodology into "classical" fields of biology and the development of theories that have the power to explain phenomena on different levels of organisation urge for the re-integration of zoological disciplines and at the same time highlight the advantages of a broader approach in the study of proximate and ultimate aspects of animal life. Zoology thus fulfils any requirements as an integrative discipline encompassing all aspects of animal life, from the level of the gene to the level of the ecosystem.

We feel that this renaissance of integrative zoology as a modern and vigorous field of research needs to be reflected in an up-to-date journal dedicated to zoology as a whole. We also believe that the best way to broadly disseminate the results of modern zoological science is provided by an Open Access, online journal. We therefore have launched *Frontiers in Zoology*, the only Open Access, online journal focussing on zoology.

## Research at the frontiers in zoology

*Frontiers in Zoology* aims at re-uniting the various sub-disciplines that investigate animal life from different angles and providing the basis for a more complete understanding of animal life. Initiated and supported by the Deutsche Zoologische Gesellschaft, one of the largest national societies devoted to zoology as a whole, and based on an editorial board () with renowned experts from different research areas, *Frontiers in Zoology* provides a novel opportunity to publish high quality articles and reviews on zoological phenomena with all the advantages provided by Open Access.

## The advantages of Open Access

As Slade et al. [1] have summarized, Open Access policy generally changes the way in which articles are published. First, all articles become freely and universally accessible online, and so an author's work can be read by anyone at no cost. Second, the authors hold copyright for their work and grant anyone the right to reproduce and disseminate the article, provided that it is correctly cited and no errors are introduced. Third, a copy of the full text of each Open Access article is permanently archived in an online repository separate from the journal. Articles in *Frontiers in Zoology* are archived in PubMed Central, the US National Library of Medicine's full-text repository of life science literature, and also in repositories at the University of Potsdam in Germany, at INIST in France and in e-Depot, the National Library of the Netherlands' digital archive of all electronic publications.

Open Access has four broad benefits for science and the general public. First, authors are assured that their work is disseminated to the widest possible audience, given that there are no barriers to access their work. This is accentuated by the authors being free to reproduce and distribute their work, for example by placing it on their institution's website. It has been suggested that free online articles are more highly cited because of their easier availability [2]. Second, the information available for researchers will not be limited by their library's budget, and the widespread availability of articles will enhance literature searching [3]. Third, the results of publicly funded research will be accessible to all taxpayers and not just those with access to a library with a subscription. Note that this public accessibility may become a legal requirement in the USA if the proposed Public Access to Science Act is made law [4]. Fourth, a country's economy will not influence its scientists' ability to access articles because resource-poor countries (and institutions) will be able to read the same materials as wealthier ones (although creating access to the internet is another matter [5]).

## The peer review policy of *Frontiers in Zoology*

Manuscripts with all figures and tables shall be submitted online using any of the listed formats (). The Editors-in-Chief or one of the Specialist Editors will forward the manuscript to two or three referees for peer-review. Upon receiving the reports from the referees, the respective Editor may suggest rejection or acceptance without, with major or with minor changes to the Editors-in-Chief, who will finalise the decision based upon the reports and the editor's opinion. Articles will be published online immediately upon acceptance and soon after listed in PubMed. Decisions about a manuscript will be exclusively based on the quality of the work, not on whether the authors can pay the article-processing charge. Note that processing charges will be waived for all authors whose institution is a BioMed Central member. Furthermore, editors can grant discretionary waivers for 35% of articles submitted in any given period of 90 days.

## Conclusion

*Frontiers in Zoology* provides an optimal way for the broad and fast dissemination of outstanding research from all areas of Zoology. We therefore invite all researchers in zoology to experience the manifold advantages provided by Open Access, online journals and to submit their high quality manuscripts to this new journal.
